# A Polymorphism Associated with Depressive Disorders Differentially Regulates Brain Derived Neurotrophic Factor Promoter IV Activity

**DOI:** 10.1016/j.biopsych.2011.11.030

**Published:** 2012-04-01

**Authors:** Benjamin Hing, Scott Davidson, Marrisa Lear, Gerome Breen, John Quinn, Peter McGuffin, Alasdair MacKenzie

**Affiliations:** a School of Medical Sciences, Institute of Medical Sciences, Foresterhill, University of Aberdeen, Aberdeen, Scotland, United Kingdom; b Institute of Psychiatry, Kings College London, London, United Kingdom; c Institute of Translational Medicine, University of Liverpool, Liverpool, United Kingdom

**Keywords:** BDNF promoter IV, brain derived neurotrophic factor, cognitive deficit and mood disorder, neuronal depolarization, single nucleotide polymorphisms, tissue specific gene regulation

## Abstract

**Background:**

Changes in brain derived neurotrophic factor (BDNF) expression have been associated with mood disorders and cognitive dysfunction. Transgenic models that overexpress or underexpress BDNF demonstrate similar deficits in cognition and mood. We explored the hypothesis that BDNF expression is controlled by balancing the activity of BDNF promoter IV (BP4) with a negative regulatory region containing a polymorphism associated with cognitive dysfunction and mood disorders.

**Methods:**

We used comparative genomics, transgenic mouse production, and magnetofection of primary neurons with luciferase reporters and signal transduction agonist treatments to identify novel polymorphic cis-regulatory regions that control BP4 activity.

**Results:**

We show that BP4 is active in the hippocampus, the cortex, and the amygdala and responds strongly to stimuli such as potassium chloride, lithium chloride, and protein kinase C agonists. We also identified a highly conserved sequence 21 kilobase 5' of BP4 that we called BE5.2, which contains rs12273363, a polymorphism associated with decreased BDNF expression, mood disorders, and cognitive decline. BE5.2 modulated the ability of BP4 to respond to different stimuli. Intriguingly, the rarer disease associated allele, BE5.2(C), acted as a significantly stronger repressor of BP4 activity than the more common BE5.2(T) allele.

**Conclusions:**

This study shows that the C allele of rs12273363, which is associated with mood disorder, modulates BP4 activity in an allele-specific manner following cell depolarization or the combined activity of protein kinase A and protein kinase C pathways. The relevance of these findings to the role of BDNF misexpression in mood disorders and cognitive decline is discussed.

Major depressive disorder (MDD) and bipolar disorder (BD) are psychiatric diseases that not only increase morbidity but also impair cognition. A gene strongly implicated in these disorders is the brain derived neurotrophic factor (BDNF). Brain derived neurotrophic factor is a neurotrophin that mediates neuroplastic processes, such as neurogenesis (1), synaptic plasticity (2), dendritic arborization (3), and development and maturation of γ-aminobutyric acid (GABA)ergic neurons (4,5). As such, alterations to BDNF expression level could impair these cellular processes affecting brain structures and their function.

Indeed, reduced BDNF expression has been observed in the hippocampus and prefrontal cortex of MDD and BD patients (6–8), coinciding with reduced volume of these structures (9–13), impaired spatial memory (14,15), and executive function (16,17), respectively. Interestingly, overexpression and underexpression of BDNF in different parts of the brain of mice impaired their learning and memory and precipitated depression-like behavior (18–20). Together, these studies highlight that fine regulation of BDNF expression level is required for mental health. This can be mediated by BDNF promoter activity.

To date, BDNF promoter IV (BP4) has been the most well-studied promoter of the nine alternative BDNF promoters and has also been shown to be important for mental health (21–25). For example, BP4 mutant mice have depression-like symptoms and impaired memory (26,27). As promoter regions are often insufficient to maintain correct messenger RNA (mRNA) expression levels in appropriate cells and in response to appropriate cues, cis-regulatory elements (CREs) are frequently required to modulate promoter activity so that transcriptional levels appropriate to health are maintained (28–31). Genetic variations, such as single nucleotide polymorphisms (SNP), in CREs can disrupt transcription factor binding sites, resulting in misregulation of gene expression causing physical changes and even disease susceptibility (32–35).

Recently, a study demonstrated that the minor allele of an SNP (rs12273363) that lies in an intergenic region upstream of the BDNF gene was associated with MDD susceptibility in patients with a history of childhood adversity (36). Considering the requirement for finely balanced levels of BDNF expression described in the studies above, we explored the hypothesis that this intergenic region where the SNP resides may be a CRE that modulates the activity of BP4 in response to specific stimuli. In addition, the SNP rs12273363 might alter the activity of this, as yet, unidentified CRE required to modulate appropriate levels of BP4 activity essential for health.

## Methods and Materials

### Bioinformatic Analysis

To identify evolutionary conserved regions (ECRs), comparative genomics was performed using the ECR genome browser (http://ecrbrowser.dcode.org/) (37) and the University of California Santa Cruz genome browser (http://genome.ucsc.edu/). Allele frequency of SNPs was determined using the National Center for Biotechnology Information Single Nucleotide Polymorphism Database (http://www.ncbi.nlm.nih.gov/SNP/).

### Plasmid Construction

Human BP4 and BE5.2 (T) were amplified using high-fidelity polymerase chain reaction (Expand High Fidelity System; Roche Diagnostics Limited, West Sussex, United Kingdom) from human DNA (Cambio, Cambridge, United Kingdom). Primers used were BP4 forward primer 5'-CTGCTCCGGGAAAGACTTC-3' and reverse primer 5'-ACCCTCGAATCACCTACCC-3'; BE5.2 forward primer 5'-GGAAATCTCGGGAAATAGGC-3' and reverse primer 5'-GACCCATCTCAGGTCTCCAG-3'. Products were ligated into pGEM-T Easy Cloning Vector (Promega, Southampton, United Kingdom). All amplified DNA samples were sequenced (Source Bioscience, Nottingham, United Kingdom) to ensure correct sequence and orientation as genomic sequence, as stated in the University of California Santa Cruz browser.

#### BP4

Brain derived neurotrophic factor promoter IV was restricted from pGEM-T Easy using NcoI and Eco53KI and cloned into pGL4.23 that had been digested with NcoI and EcoRV (Promega) to replace the minimal promoter.

#### BP4 BE5.2(T)

Brain derived neurotrophic factor promoter IV was restricted from pGEM T Easy using NcoI and SpeI, while BE5.2 was restricted using SpeI and ZraI. They were subsequently ligated into pGL4.23, which had been digested with NcoI and EcoRV to remove the minimal promoter.

#### BP4 BE5.2(C)

The major allele T was substituted with the minor allele C by site-directed mutagenesis using QuickChange II site-directed mutagenesis kit (Agilent Technologies, Cheadle, United Kingdom). Primers used were forward primer 5'-ACAGCGATGCTGCAGAAGA**C**GGTGGATAGCTC-3' and reverse primer 5'-GAGCTATCCACC**G**TCTTCTGCAGCATCGCTGT-3'.

#### ΔBP4

To control for cell-specific or treatment effects on the pGL4 plasmid backbone, we produced a plasmid identical to the pGL4.23 BP4 plasmid but that lacked BP4. The minimal promoter was removed from pGL4.23 using NcoI and Bgl II and subsequently blunt-ended using DNA polymerase I large Klenow fragment before ligation.

#### BP4 LacZ

Brain derived neurotrophic factor promoter IV was removed from pGEM T Easy using NcoI and EcoICRI (Promega) and ligated into the SphI (made blunt end using Klenow fragment) and NcoI site of the p1230 plasmid (a kind gift from Robert Krumlauf, Stowers Institute, Kansas City, Missouri) that encodes a LacZ marker.

### Primary Cell Culture

Hippocampal, cortex, and amygdala tissues, as previously defined (38), were dissected from postnatal day 0 to day 3 wild-type Sprague Dawley rat pups that were humanely sacrificed according to United Kingdom Home Office guidelines. Tissues were treated with .05% trypsin ethylenediaminetetraacetic acid (Invitrogen, Paisley, Scotland, United Kingdom) for 15 minutes at 37°C. Trypsin ethylenediaminetetraacetic acid was replaced with soybean trypsin inhibitor (Sigma, Irvine, Scotland, United Kingdom) for 5 minutes at room temperature to stop reaction. This was then replaced with unsupplemented Neurobasal A (Invitrogen) followed by mechanical dissociation. Cells were then resuspended in culture media (Neurobasal A, B27 [Invitrogen], 1X GlutaMAX [Invitrogen], and Pen/strep [100 U/mL] [Invitrogen]) and plated out at a density of ∼80,000 viable cells/cm^2^ on Poly-L-Lysine (20 μg/mL) (Sigma) pretreated plates following cell counting for viable cells using TC10 automated cell counter (Biorad, Hemel Hempstead, United Kingdom) with trypan blue. Cells were incubated at 37°C, 5% carbon dioxide for 7 days before transfection.

### Transfections and Treatments

All DNA constructs were quantified on a nanodrop machine (NanoDrop Technologies, Thermo Scientific, Southend-on-Sea, United Kingdom). Quantities of plasmid used for each transfection were adjusted to the size of each plasmid to ensure molar equivalence between experiments. Firefly luciferase plasmids were co-transfected with Renilla luciferase plasmid, pGL4.70 (Promega), to normalize signals between transfections using magnetic particles (Neuromag; Oz Bioscience, United Kingdom) as described in manufacturer's instructions. Primary neuronal cultures were incubated for 24 hours before agonist or vehicle treatment. Stock potassium chloride (KCl) and lithium chloride (LiCl) treatments were prepared in culture media, whereas phorbol-12-myristate-13-acetate (PMA) was prepared in dimethyl sulfoxide or ethanol. Forskolin (Sigma) was prepared in dimethyl sulfoxide (Sigma). All treatments were diluted in culture media to the following concentrations for use unless otherwise stated: 60 mmol/L KCl, 1 mmol/L LiCl, 150 nmol/L PMA, 25 μmol/L forskolin. Cultures treated with KCl, PMA, and forskolin were incubated for 24 hours before harvest for dual luciferase assay. To maintain consistency with previous studies, cultures treated with 1 mmol/L LiCl were incubated for 72 hours as previously described (39) before harvest for dual luciferase assay.

### Dual Luciferase Assay

Cells were harvested and dual luciferase assays were performed as described in manufacturer's instructions using GloMax 96 Microplate Luminometer with dual injectors (Promega).

### Transgenic Mouse Production

Transgenic mice were generated as previously described (40).

### Analysis of LacZ Gene Expression in Transgenic Lines

Neonate (2–7 days old) F1 transgenic mouse brains were fixed and stained as previously described (41). X-Gal (Sigma) stained tissues were then prepared for vibratome sectioning as previously described (42).

### Data Analysis

All experiments were performed at least three independent times in triplicate (i.e., *n* ≥ 3). Statistical significance of data sets was analyzed using either two-way analysis of variance with post hoc Holm-Sidak test using SigmaPlot Build 11.0.0.75 (Systat Software Inc., Hounslow, United Kingdom) or two-tailed unpaired parametric Student *t* test using GraphPad PRISM version 5.02 (GraphPad Software, La Jolla, California), where appropriate.

## Results

### BP4 is Active in the Amygdala, Hippocampus, and Cortex of Transgenic Mice

**Figure 1:**
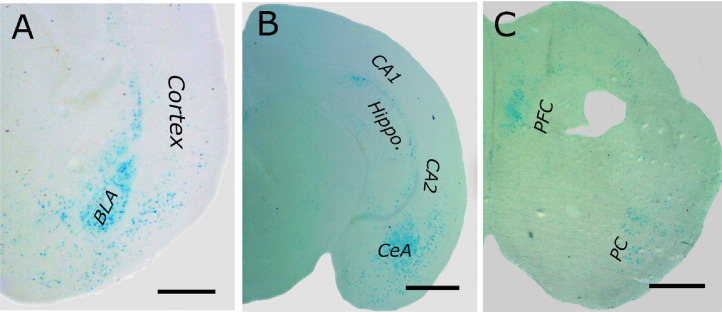
Brain derived neurotrophic factor promoter 4 can support LacZ expression in cortex, hippocampus, and amygdala. **(A–C)** Photomicrographs of 60-μm sections of brain derived neurotrophic factor promoter 4-LacZ neonatal (postnatal days 3–7) transgenic mouse brains following staining by X-Gal showing LacZ expression (blue cells) in the amygdala **(A)**, hippocampus **(B)**, and prefrontal cortex **(C)**. Scale bars represent 500 μm **(A)**, 1000 μm **(B)**, and 880 μm **(C)**, respectively. BLA, basolateral amygdala; CA, cornu ammonis; CeA, central amygdala; Hippo, hippocampus; PC, piriform cortex; PFC, prefrontal cortex.

Although a great deal has been discovered about the role of BP4 in the brain and behavior (21,26,27), little is known about the tissue-specific activity of BP4 in vivo. To address this deficit, three transgenic mouse lines were created that expressed the LacZ gene under the control of human BP4. Coronal brain sections derived from these transgenic lines showed consistent X-Gal staining present in structures in the hippocampus, prefrontal cortex, and amygdala (Figure 1A–C). These expression patterns produced by the BP4-LacZ transgene closely resembled endogenous mouse BDNF exon IX mRNA distribution documented in the Allen Institute for Brain Science Brain Atlas (http://www.brain-map.org/).

### Activity of BP4 in Untreated Primary Hippocampal, Cortical, and Amygdala Cultures

**Figure 2:**
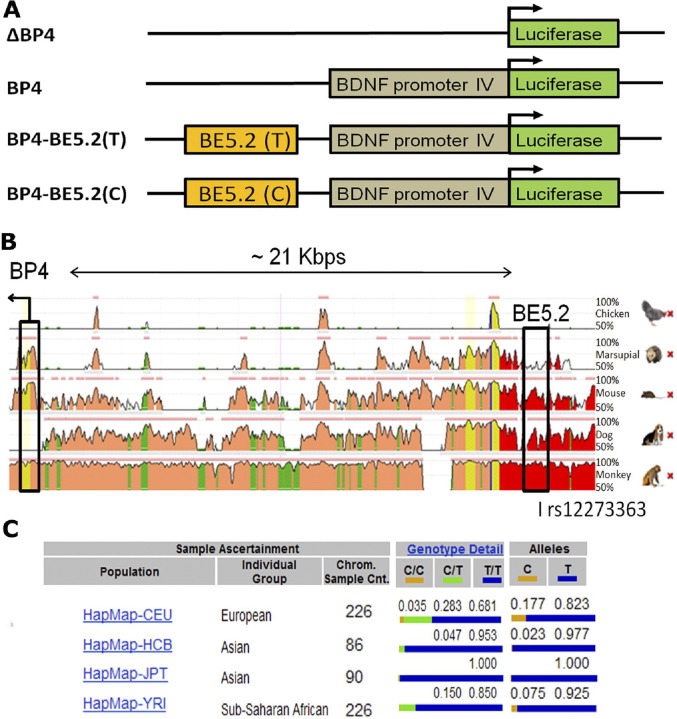
Diagrams representing luciferase constructs used for the investigation and comparative genomic analysis to identify BE5.2 and allelic frequency of rs12273363 in different human populations. **(A)** Diagrammatic representation of the different luciferase constructs used to investigate the effects of different alleles of BE5.2 and on human brain derived neurotrophic factor promoter 4 (BP4) activity (not to scale). **(B)** Stacked pairwise alignment from the evolutionary conserved region genome browser between genomic sequences of different animal species (in descending order; chicken, possum, mouse, dog, and rhesus macaque) against the human genomic sequence. Brain derived neurotrophic factor promoter 4 and BE5.2 are indicated by a black box and the direction of transcription from BP4 is indicated by a bent black arrow. The single nucleotide polymorphism rs12273363 is found in BE5.2. Yellow, pink, green, blue, and red peaks highlight areas of conservation in untranslated regions, introns, repetitive regions, coding regions, and intergenic DNA, respectively (from evolutionary conserved region genome browser). The position of rs12273363 is indicated below BE5.2. **(C)** Allelic frequency of rs12273363 in different human populations from the National Center for Biotechnology Information single nucleotide polymorphism database. BDNF, brain derived neurotrophic factor; CEU, Central Europeans; Chrom., chromosome; Cnt., count; HCB, Hans Chinese in Bejing; JPT, Japanese in Tokyo; YRI, Yoruba in Ibadan.

**Figure 3:**
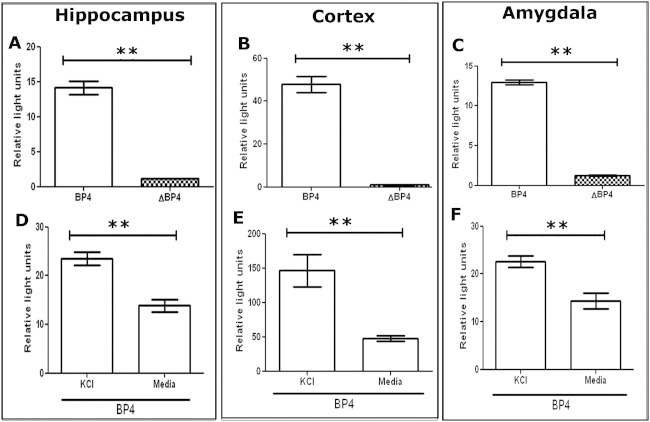
Brain derived neurotrophic factor promoter 4 (BP4) and brain derived neurotrophic factor activity in primary neuronal cultures following potassium chloride (KCl) depolarization. Primary hippocampal **(A, D)** cortical **(B, E),** and amygdala **(C, F)** cultures were co-transfected with BP4 and renilla plasmid. Cells were either untreated **(A–C)** or treated **(D–F)** with 60 mmol/L KCl for 24 hours before harvesting for analysis by dual luciferase assay. Relative light units were calculated by normalizing luciferase signal to renilla signal. *n* ≥ 3, **p < .01.

To investigate BP4 activity in primary cell culture, BP4 was cloned into a luciferase plasmid (Figure 2A) and transfected into primary neuronal cultures. BP4 induced significantly higher luciferase activity than the control plasmid, ΔBP4, in primary hippocampal cultures (Figure 3A), cortical cultures (Figure 3B), and amygdala cultures (Figure 3C).

### KCl Depolarization Upregulates Both Endogenous BDNF Gene Expression and BP4 Activity in Primary Hippocampal, Cortical, and Amygdala Cultures

In previous studies, BP4 activity was observed to be upregulated following neuronal depolarization using KCl (23,24,43). We therefore investigated whether endogenous BDNF gene expression or BP4 responded to KCl depolarization in our primary cell paradigms. Consistent with these previous studies, we observed that KCl significantly increased endogenous rat BDNF exon IV and exon XI (data not shown). This coincided with a significant increase in BP4 luciferase activity in hippocampal (Figure 3D), cortical (Figure 3E), and amygdala cultures (Figure 3F) compared with untreated cultures. These results demonstrate parity of our current primary cell model system with previous studies (23,24,43). The ΔBP4 did not respond to KCl depolarization in any of the cell types studied (data not shown).

### Comparative Genomics Identified a Novel Polymorphic Putative Cis-regulatory Element Upstream of Human BP4

In concert with previous studies, we have shown that BP4 is active in specific parts of the brain and responds to KCl depolarization (22,44–46). As no SNPs to date have been identified in BP4 that affects its activity or associates with disease, we hypothesized that a second regulatory element in the form of a CRE harbors disease-related genetic mutation in and around the BDNF locus. We have previously shown that comparative genomics is a powerful method to identify novel CREs that may lie some distance from the promoter regions they affect (40,42,47–49). Using the ECR genome browser, we identified a highly conserved ECR that lay ∼21 kilobase pairs upstream of BP4 in an intergenic region (Figure 2B). This ECR (BE5.2) was highly conserved (75.9% similarity over 793 base pair) in placental mammals representing 92 million years of divergent evolution (50) and coincided with the location of an SNP, rs12273363, that had previously been associated with a number of conditions including MDD and BD (36,51) and resulted in a T-C change within BE5.2. Since BP4 is active in the hippocampus, cortex, and amygdala, the effects of the two different alleles of BE5.2 on BP4 activity were investigated in primary neuronal cultures derived from these brain structures. From the Single Nucleotide Polymorphism Database (http://www.ncbi.nlm.nih.gov/SNP/), the allele T is the major allele, while the allele C is the minor allele in all investigated human populations (Figure 2C). As such, BE5.2 containing the major allele T or minor allele C will be termed BE5.2(T) and BE5.2(C), respectively, for the rest of the investigation.

### BE5.2(T) Reduces BP4 Activity in Untreated Primary Hippocampal, Cortical, and Amygdala Cultures but Permits Upregulation of BP4 Activity by KCl

**Figure 4:**
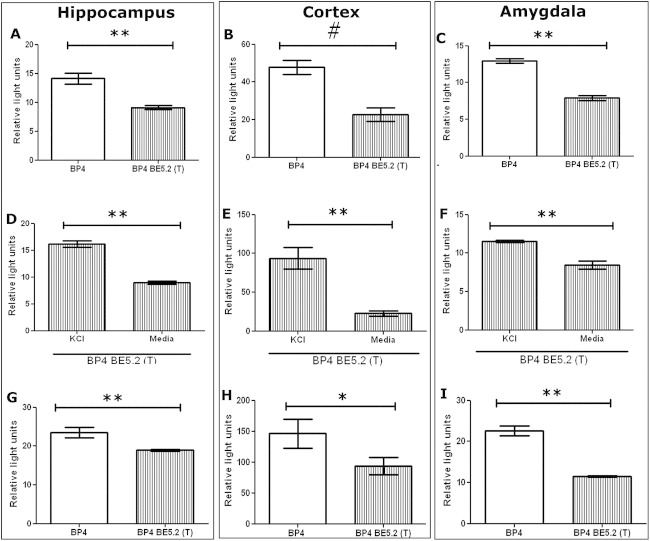
Brain derived neurotrophic factor promoter 4 (BP4) and BP4-BE5.2(T) response to potassium chloride (KCl) depolarization in primary neuronal cultures. Primary hippocampal **(A, D, E)**, cortical **(B, E, H),** and amygdala **(C, F, I)** cultures were co-transfected with BP4 or BP4-BE5.2(T) and renilla plasmid. Cells were either untreated **(A–C)** or treated **(D–I)** with 60 mmol/L KCl for 24 hours before harvesting for analysis by dual luciferase assay. Relative light units were calculated by normalizing luciferase signal to renilla signal. *n* ≥ 3, **p < .01, *p < .05, not significant; ^#^two-way analysis of variance *p* > .05 but *t* test *p* < .01.

To investigate the effect of BE5.2(T) on BP4, BE5.2(T) was cloned into a luciferase plasmid upstream of BP4 (Figure 3A) and transfected into three different primary neuronal cultures. BP4-BE5.2(T) reduced luciferase activity compared with BP4 in all untreated primary cell cultures, suggesting that BE5.2 acts to suppress BP4 activity (Figure 4A–C).

Although the response of BP4 was reduced by BE5.2(T) (Figure 4G–I), BE5.2(T) permitted activation of BP4 by KCl treatment in all primary cell cultures used (Figure 4D–F).

### BE5.2 Demonstrates Significant Allele-Specific Differences in its Ability to Reduce BP4 Response to KCl Depolarization in Primary Hippocampal and Amygdala Cultures

**Figure 5:**
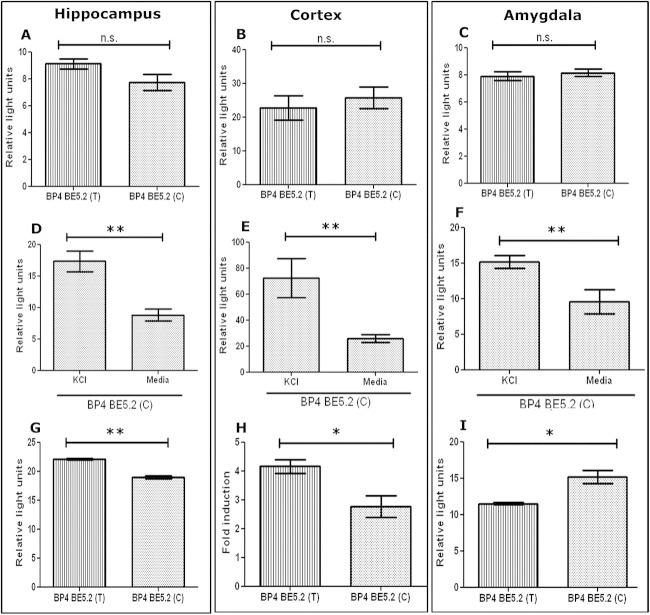
Comparison of BE5.2(T) and BE5.2(C) interaction with brain derived neurotrophic factor promoter 4 (BP4) in potassium chloride (KCl) depolarized hippocampal, cortical, and amygdala cultures. Primary hippocampal **(A, D),** cortical **(B, E),** and amygdala **(C, F)** cultures were co-transfected with either BP4-BE5.2(T) or BP4-BE5.2(C) with renilla plasmid. Cells were untreated **(A–C)** or treated **(D–I)** with 60 mmol/L KCl for 24 hours before harvest for dual luciferase assay. Relative light units were calculated by normalizing luciferase signal to renilla signal. Fold induction was calculated by dividing relative light units of treated culture by relative light units of vehicle-treated cultures. *n* ≥ 3, **p < .01, *p < .05. n.s., not significant.

To detect functional differences between the two alleles of BE5.2, primary neuronal cultures were transfected with BP4-BE5.2(T) and BP4-BE5.2(C). In all untreated primary neuronal cultures, no significant difference in luciferase activity was observed between BP4-BE5.2(T) and BP4-BE5.2(C) (Figure 5A–C). Both BP4-BE5.2(T) (Figure 4D–F) and BP4-BE5.2(C) responded to KCl in hippocampal (Figure 5D), cortical (Figure 5E), and amygdala (Figure 5F) cultures. However, BE5.2(C) did not permit BP4 to respond as strongly as BE5.2(T) to KCl depolarization in primary hippocampal (Figure 5G) and cortical cultures (Figure 5H). By contrast, in amygdala cultures, BP4-BE5.2(C) had significantly increased response to KCl compared with BP4-BE5.2(T) (Figure 5I).

### BE5.2 Abolished BP4 Response to LiCl in Primary Cortical Cultures

**Figure 6:**
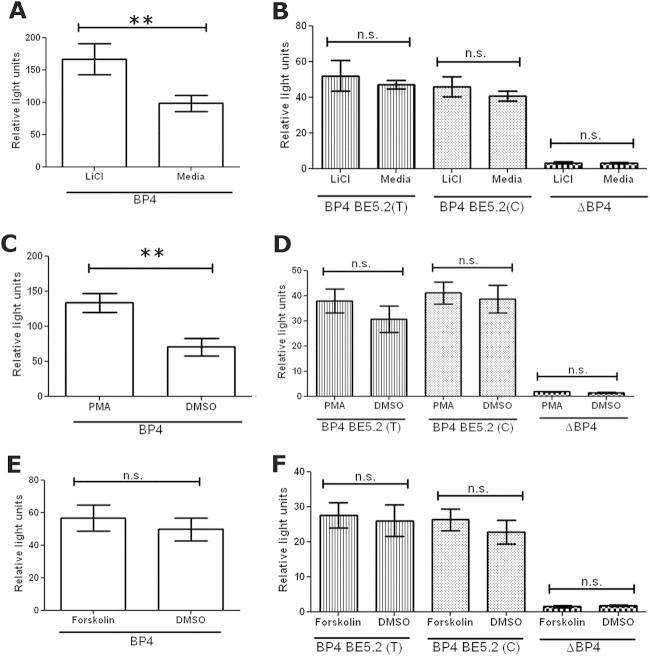
Interaction of BE5.2 (C and T) with brain derived neurotrophic factor promoter 4 (BP4) in response to lithium chloride (LiCl), phorbol-12-myristate-13-acetate (PMA), and forskolin in primary cortical cultures. Primary cortical cultures were co-transfected with either BP4 **(A, C, D)** or BP4-BE5.2(T), BP4-BE5.2(C), or ΔBP4 **(B, D, E)** with renilla plasmid. Cells were treated with 1 mmol/L LiCl for 72 hours **(A, B)**, 150 nmol/L PMA for 24 hours **(C, D),** or forskolin for 24 hours **(E, F)** before harvest for dual luciferase assay. Relative light units were calculated by normalizing luciferase signal to renilla signal. *n* ≥ 3, **p < .01. DMSO, dimethyl sulfoxide; n.s., not significant.

Recently, the established antidepressant and Wnt agonist LiCl was shown to increase rat BP4 activity in primary cortical cultures (39). We, therefore, investigated the effect of the different alleles of BE5.2 on BP4 response to Wnt signaling in primary cortical cultures. Consistent with previous studies, LiCl significantly increased BP4 activity in primary cortical cultures (Figure 6A). However, in the presence of either BE5.2(C) or BE5.2(T), BP4 activation by LiCl was abolished (Figure 6B). No significant allele-specific differences were observed.

### BE5.2 Prevents Increase of BP4 Activity by PMA and Forskolin

It has been previously shown that KCl activates the protein kinase C (PKC) (52,53) and protein kinase A (PKA) pathways (54–57), which have been shown to increase BDNF exon IV expression (58,59). We, therefore, investigated if either of these signaling pathways acted on BP4 and whether BE5.2 modulated the BP4 response in any way. First of all, cortical cultures were treated with PMA, an agonist of the PKC pathway (60). Phorbol-12-myristate-13-acetate significantly increased BP4 activity (Figure 6C). However, both BE5.2(T) and BE5.2(C) abolished the ability of BP4 to respond to PMA (Figure 6D). Moreover, treatment with forskolin did not significantly upregulate activity of BP4 and neither BE5.2(T) nor BE5.2(C) changed this response significantly (Figure 6E,F).

### BE5.2(T) and BE5.2(C) Display Significant Allele-Specific Differences in Their Ability to Suppress BP4 Activity Following Combined Stimulation by PKC and PKA Pathways

**Figure 7:**
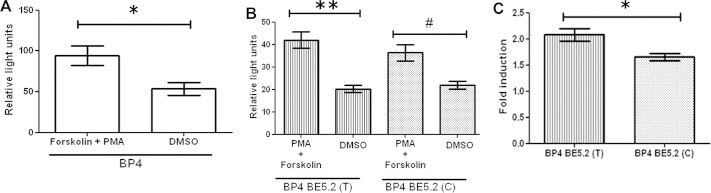
Combined treatment of phorbol-12-myristate-13-acetate (PMA) and forskolin increased luciferase expression by brain derived neurotrophic factor promoter 4 (BP4) BE5.2 (T) and BP4 BE5.2 (C) in cortical cultures. Primary cortical cultures were co-transfected with either **(A)** BP4 or **(B, C)** BP4 BE5.2(T) and BP4 BE5.2(C). Cells were treated with 150 nmol/L PMA and 25 μmol/L forskolin or dimethyl sulfoxide (DMSO) as vehicle treatment for 24 hours before harvest for dual luciferase assay. Relative light units were calculated by normalizing luciferase signal to renilla signal. Fold induction was calculated by dividing relative light units of treated culture by relative light units of vehicle-treated cultures. *n* ≥ 3, **p < .01, *p < .05, and ^#^two-way analysis of variance *p* > .05 but *t* test *p* < .05.

Because KCl depolarization is known to alter BDNF gene expression through activation of both PKA and PKC pathways, we investigated the hypothesis that activation of BP4 by KCl required a combination of both these pathways. When transfected cortical cultures were treated with both PMA and forskolin, BP4 activity was significantly increased (Figure 7A). However, in contrast to our previous observations, whereby BE5.2 abolished activation of BP4 by PMA, BE5.2 permitted PMA activation of BP4 in the presence of both forskolin and PMA (Figure 7A,B). This suggests that PKC and PKA pathways work synergistically through BE5.2 to permit BP4 activation by PMA. Significantly, we were also able to observe that BE5.2(C) was less permissive of BP4 activation by PMA and forskolin than BE5.2(T) (Figure 7B,C). These observations are consistent with our previous observations of differential allelic response following KCl depolarization (Figure 5H).

## Discussion

In the current study, we have shown that human BP4 is active in cells of the prefrontal cortex, cortex, hippocampus, and amygdala in a pattern that mirrors the expression pattern of endogenous mouse BDNF mRNA (22,44–46). These results confirm previous studies using transgenic mice carrying a bacterial artificial chromosome containing the human BDNF locus that also showed BDNF expression in these regions (45). However, these bacterial artificial chromosome-based studies could not attribute the expression patterns produced directly to BP4. In a separate study using human tissues, reverse transcription polymerase chain reaction also showed BDNF exon IV expression in prefrontal cortex, cortex, hippocampus, and amygdala (21). This study was, however, unable to demonstrate that BP4 itself was sufficient to direct BDNF exon IV expression in these brain structures independent of CREs, since CREs are known to be able to direct the spatial activity of promoters (61,62). To circumvent this problem, we expressed LacZ under the regulation of BP4 in mice and demonstrated, for the first time, that human BP4 is active in brain structures that express BDNF mRNA and are known to be altered in MDD and BD (10–13,63,64). In addition, the current study demonstrates that BP4 is a highly active positive regulatory element whose activity is further increased by a number of different signal transduction pathways. However, the possibility that BP4 is solely responsible for controlling BDNF expression is inconsistent with previous studies demonstrating that overexpression or underexpression of BDNF affects behavior and cognition in a similar way (18,20). Considering the requirement for a fine balancing of BDNF expression levels in health and the strong positive role of BP4 in activating gene expression (analogous to an accelerator), we explored the hypothesis that some type of balancing negative regulator may be required to allow appropriate control of BDNF levels (i.e., a brake).

Many studies have reported examples of negative regulation (61,65). Indeed, SNPs in silencer elements have been observed to contribute to disease and anxiety traits (66–68). Silencer elements have been known to not only reduce promoter activity but to also abolish promoter response to stimuli (69–71). In line with this, our study identified a highly conserved CRE called BE5.2, which contained a polymorphism associated with mood disorders that reduced BP4 activity in all our primary neuronal cultures and abolished BP4 response to PKC activation by PMA treatment. Intriguingly, BE5.2 permitted BP4 response to KCl depolarization, although at lower levels, which is a novel and interesting property of silencer elements never observed before. Although BE5.2 blocked BP4 activation by the PKC pathway, we eventually deduced that BE5.2 permitted activation of BP4 by PKC but only if the PKA pathway was also activated. Based on these observations, we propose the hypothesis that BE5.2 does not act as an indiscriminate silencer but rather filters signaling information appropriate for normal BP4 response to neuron activation. Our data suggest that this information filtering system selectively favors BDNF gene expression mediated by KCl depolarization through activation of PKA and PKC pathways, ensuring that other ligand-receptor interactions, which induce only PKC or Wnt signaling pathways, are less likely to trigger inappropriate expression of the BDNF gene.

In addition to presenting data supporting the role of BE5.2 as a filter of signaling information, our observations suggested that in hippocampal and cortical cultures, BE5.2(C) was a stronger silencer of BP4 activity than BE5.2(T), as it reduced the ability of BP4 to respond to neuron activation by KCl depolarization. This observation is consistent with a possible role of the C allele in further reducing BP4 response to neuron activation by KCl depolarization with probable consequences for BDNF mRNA and protein levels in these cell types. Consistent with this observation, a postmortem study on brain tissues derived from human MDD and BD patients who were homozygous or heterozygous for the C allele of rs12273363 had significantly reduced hippocampal BDNF levels compared with those who were homozygous for the major allele (72). Indeed, decreased BDNF levels in the hippocampus and prefrontal cortex of MDD and BD patients are well described in postmortem studies (6,8,73). Taken together, our present study, when examined in the light of these previous observations (72), is consistent with the hypothesis that the C allele reduces BDNF expression level by reducing BP4 activity to neuron activation, thus contributing to susceptibility to mood disorders.

In contrast to hippocampal and cortical cultures, the minor allele of rs12273363 reduced the suppression effect of BE5.2 in amygdala cultures, increasing BP4 response to neuron activation by KCl depolarization. These observations are consistent with reports of SNPs with tissue-specific effects, suggesting that regulatory SNPs can affect promoter activity in a context-dependent manner (74,75).

Any increase in BP4 activity could hypothetically increase BDNF expression in the amygdala, thus increasing neuroplastic processes including dendritic and axonic arborization (76,77), neurogenesis (1,78) and GABAergic neuron development (79,80). Although neurogenesis is commonly known to occur in the dentate gyrus of the hippocampus (81,82) and the subventricular zone (83), studies have also suggested that it might also occur in the amygdala (84,85). Since the amygdala is involved in aversive fear response and emotional memory, increase in these cellular processes in the amygdala may contribute to anxiety behavior and poorer emotional memory as observed in depressive disorder patients with enlarged amygdala (86,87). Placing our data in the light of these previous studies, it is possible that increased BDNF promoter IV activity by the minor C allele of rs12273363 in the amygdala might increase these cellular processes with consequences for anxiety behavior and emotional memory.
